# Daily Relationships Between Physical Activity and Sleep: Differences Between Subjective and Objective Measures

**DOI:** 10.1093/geroni/igaa057.1376

**Published:** 2020-12-16

**Authors:** Alycia Bisson, Margie Lachman, Alycia Sullivan Bisson

**Affiliations:** Brandeis University, Waltham, Massachusetts, United States

## Abstract

Although there is evidence that physical activity (PA) and sleep are related, it is unclear which aspects of these multidimensional constructs are involved. Many have examined differences in PA and sleep between persons, but few have tested daily associations within persons. The present study examined sleep (duration; hours spent asleep, WASO; wake after sleep onset, latency; time to fall asleep) and PA (total and intensity) over 7 days, using both a self-reported diary (subjective) and an ActiWatch (objective). Healthy adults between 34 and 83 came to University of Wisconsin, Madison to participate in the Midlife in the United States (MIDUS) Biomarker study (N=436, Mage: 56.92, SDage: 11.5). Subjective and objective measures showed differential relationships; subjective duration was higher, and latency was lower than objective measures. Some age differences were also found; older adults reported more WASO than middle-aged adults, but their WASO was similar according to actigraphy. Multilevel models revealed that total PA and intensity significantly predicted subjective and objective sleep measures, controlling for age, sex, and other demographic variables. More active participants had shorter sleep durations, WASO, and latency. Within-person analyses revealed that on days one is more active than average, sleep duration is shorter with less WASO across age. Although the negative relationship between PA and sleep duration was unexpected, it is possible that because more active individuals wake less during the night, they may need fewer hours of sleep because their sleep is more restful. Discussion will focus on possible mechanisms involved in linking PA and sleep.

